# Calcified Canal Conquered: A Case Report of Minimally Invasive Management of a Calcified Maxillary Central Incisor Using the PriciGuide® System

**DOI:** 10.7759/cureus.72743

**Published:** 2024-10-30

**Authors:** Ashwija Shetty, Hajira A Sultana, Keerthan B V, Varun Prabhuji

**Affiliations:** 1 Department of Conservative Dentistry and Endodontics, The Oxford Dental College, Bengaluru, IND

**Keywords:** calcified canals, cone beam computed tomography, guided endodontics, minimally invasive, precision, root canal treatment

## Abstract

Calcification of the root canal system presents a significant clinical challenge, increasing the risk of procedural complications. This case report describes the management of a 26-year-old female patient who presented with a chief concern of discoloration in her maxillary central incisors. Radiographic examination revealed a periapical lesion and obliteration of the canal in the left maxillary central incisor. The case highlights the effective use of the PriciGuide® system in accurately locating the calcified canal while preserving the integrity of a healthy tooth structure. Following root canal treatment, aesthetic rehabilitation was performed, which included internal bleaching and the fabrication of direct composite veneers. This guided endodontic approach enabled safe negotiation of the calcified canal, ensuring thorough cleaning, shaping, and filling. By minimizing unnecessary dentin removal, the PriciGuide® system provided a conservative solution that contributed to the preservation of tooth integrity and the successful treatment of this challenging case.

## Introduction

Pulp canal obliteration, often referred to as calcific metamorphosis, is a frequent outcome of dental trauma. The pulp's reaction to trauma leads to a calcification of the root canal [1]. Periapical bone lesions were observed in 7-27% of cases with pulp canal obliteration. While root canal treatment is generally recommended for teeth exhibiting radiographic signs of periapical disease. However, it can pose significant challenges in cases of pulp canal obliteration, as determining the exact position of the root canal is often problematic [2].

A meticulously planned and guided minimally invasive access cavity can help preserve tooth structure and prevent perforations, potentially leading to a better long-term outcome, especially for teeth with calcified root canals [3]. A significant limitation of the existing sleeve-guided system is its tendency to deviate, restrict visibility and impede coolant flow to the bur, leading to potential overheating [4].

The PriciGuide® system, a sleeve-free design that uses guide rails to position the bur, offers improved visibility, reduces deviation, and ensures adequate coolant flow to prevent bur overheating. By enabling dentists to achieve optimal access for root canal treatment while minimizing dentin loss, the PriciGuide® system contributes to the advancement of minimally invasive endodontics, ultimately improving long-term outcomes for patients with pulp canal obliteration [5].

This case report introduces an innovative guided endodontic technique utilizing the PriciGuide® system. The system was used to access an obliterated root canal in a maxillary central incisor with a periapical lesion. The case was followed by aesthetic rehabilitation involving internal bleaching and direct composite veneers.

## Case presentation

**Figure 1:**
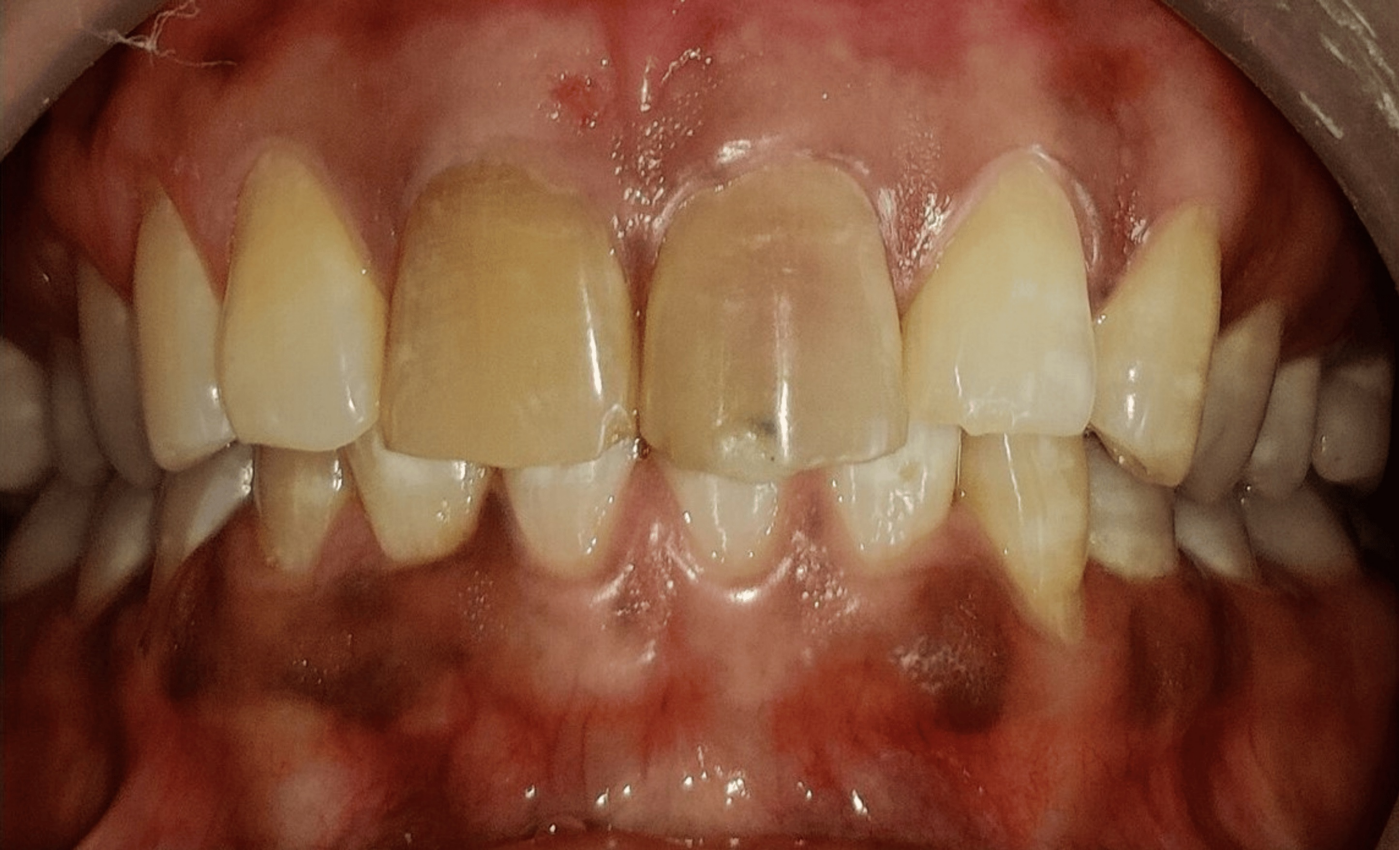
Pre-operative clinical image

A 26-year-old female patient visited the department, reporting a five-year history of discoloration in her maxillary anterior teeth. The patient reported a history of trauma to the teeth 12 years ago. Clinical examination revealed discolored maxillary central incisors (Figure 1).

**Figure 2:**
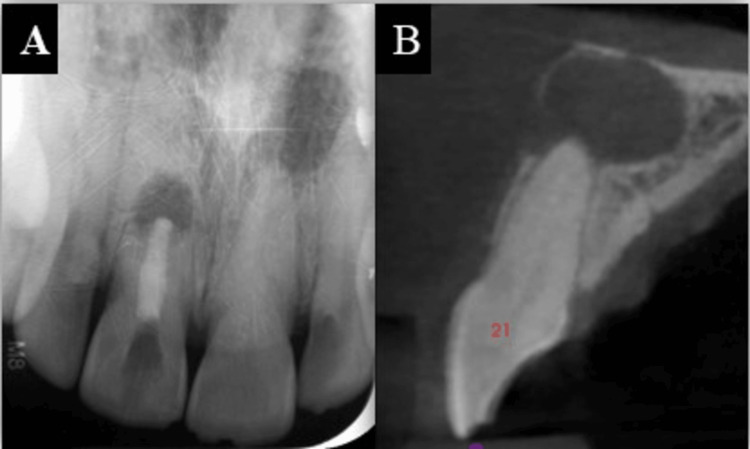
(A) Pre-operative radiograph and (B) pre-operative CBCT image CBCT: Cone beam computed tomography

Pulp sensibility tests were non-responsive. Tooth #21 was grade 1 mobile and tender to vertical percussion. Radiographic evaluation showed a root canal-treated tooth #11 and a calcified canal with a periapical lesion in tooth #21 (Figure 2A). Based on the patient's clinical presentation and radiographic examination, tooth #21 was diagnosed with a chronic periapical abscess.

The PriciGuide® system (Roots to Cusps® Private Limited, Bengaluru, India) was selected for access opening in the calcified canal due to its simplicity and ease of use. A cone beam computed tomography scan was performed to obtain the digital imaging and communications in medicine (DICOM) file and an impression was taken (Figure 2B).

**Figure 3:**
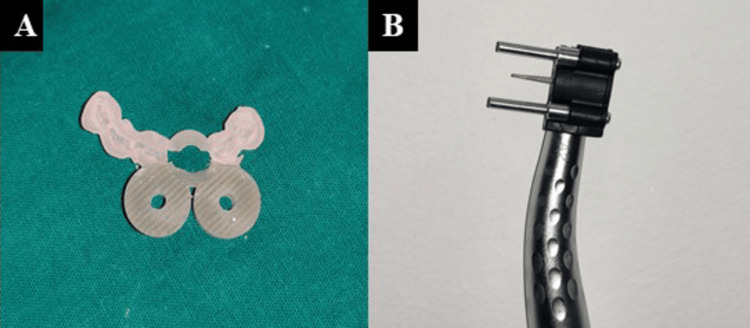
(A) Customised sleeveless guide and (B) PriciGuide® system PriciGuide® system: Roots to Cusps® Private Limited, Bengaluru, India

This was digitized into a standard tessellation language (STL) file using a laboratory scanner. Both the DICOM and STL data were sent to Roots to Cusps® Private Limited for the fabrication of a patient-specific guide for the tooth (Figures 3A, 3B) [6].

**Figure 4:**
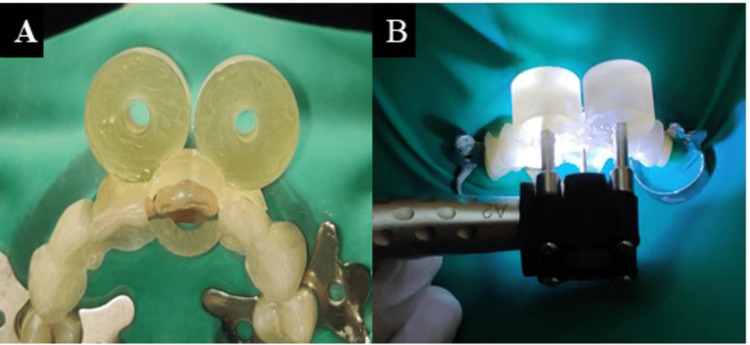
(A) Guide positioned on the patient’s teeth and (B) access cavity created with the PriciGuide® system PriciGuide® system: Roots to Cusps® Private Limited, Bengaluru, India

The guide was then positioned on the patient’s teeth and using a specialized attachment to the airotor, the access cavity was created for tooth (Figures 4A, 4B).

**Figure 5:**
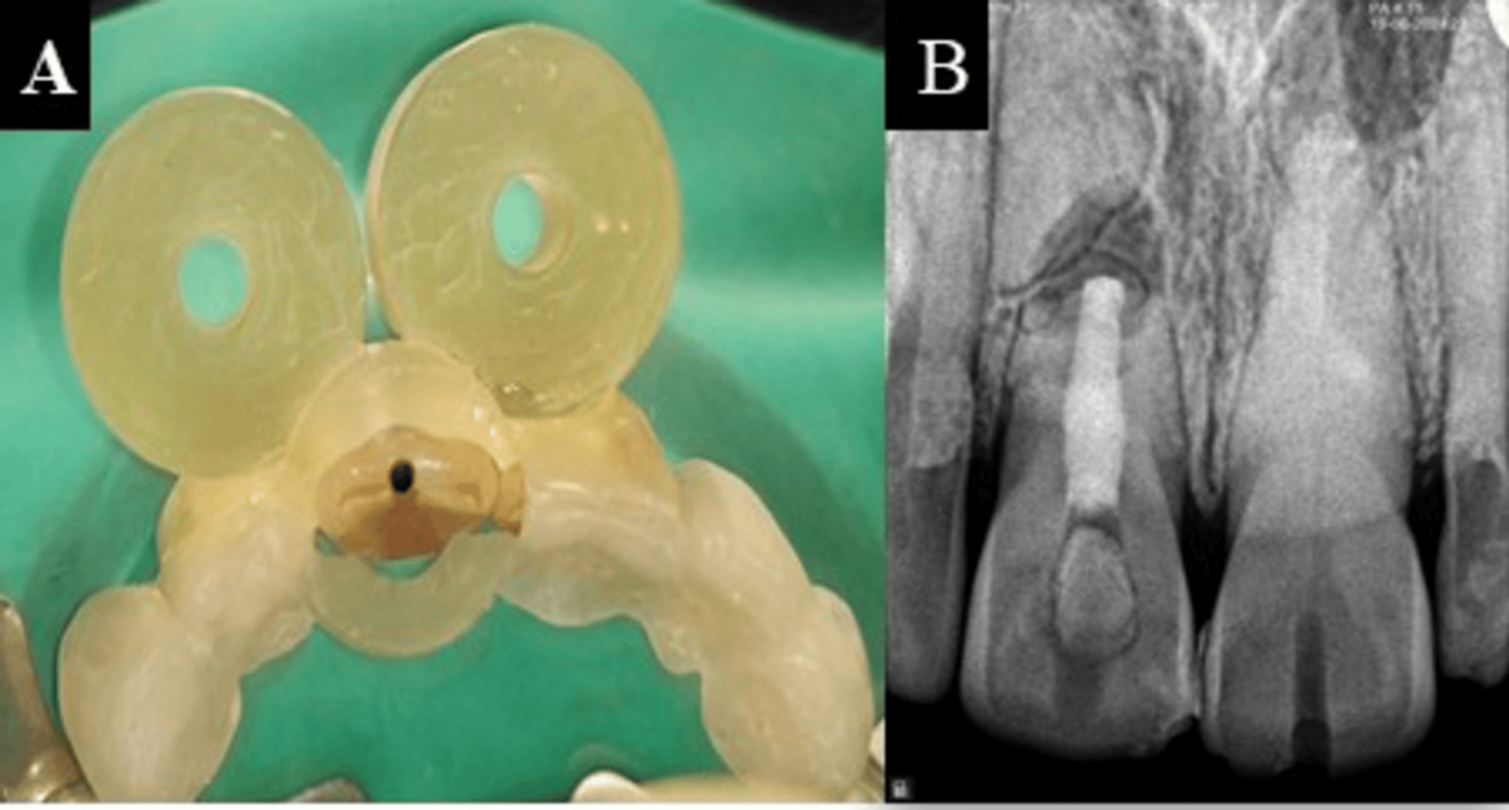
(A) Minimally invasive access opening on the tooth and (B) minimally invasive access opening radiograph

Following the access opening, the canal was irrigated with Twin Kleen and negotiated with C-files (Figures 5A, 5B).

**Figure 6:**
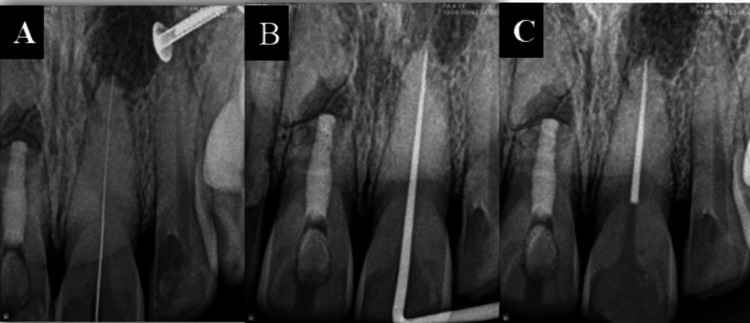
(A) Working length radiograph, (B) master cone verification radiograph and (C) obturation radiograph

The working length was determined using an apex locator and confirmed with digital radiographs (Figure 6A). The canal was shaped to a size of 30/4%. Double antibiotic paste was placed as an intracanal medicament and the access cavity was temporized.

At the subsequent visit, there was no mobility or tenderness to percussion. The canal was obturated using a single cone technique with a bioceramic sealer (Figures 6B, 6C).

**Figure 7:**
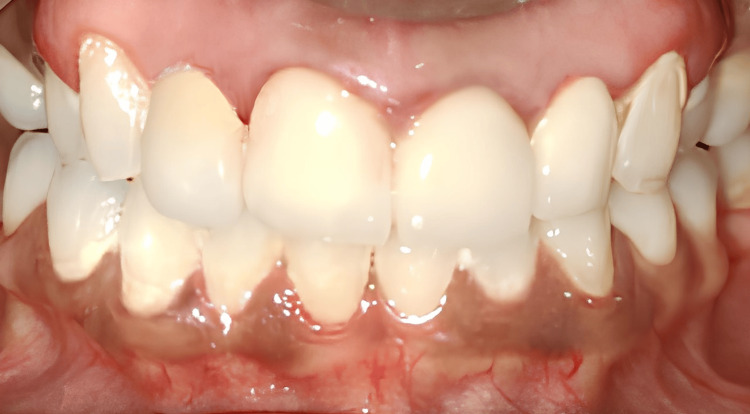
Direct composite veneers

The patient was recalled after a week for internal bleaching with Opalescence Endo. However, as the results were unsatisfactory, further aesthetic enhancement was achieved with direct composite veneers (Figure 7).

## Discussion

Pulp calcification often manifests as tooth discoloration, occurring in approximately 70-80% of cases. This discoloration is due to the loss of translucency due to dentin formation within the pulp chamber. Since pulp calcification due to trauma often affects aesthetically important teeth, various treatment options have been developed. These include external or vital bleaching, internal and external bleaching without root canal treatment, internal bleaching with root canal treatment, and prosthetic restorations [7].

In this case, a conservative approach was chosen, consisting of root canal treatment followed by aesthetic rehabilitation using internal bleaching and direct composite veneers.

Root canal location and preparation in teeth with pulp canal obliteration can be challenging, even with the aid of an operating microscope. Potential procedural errors include excessive access cavity preparation, iatrogenic perforations, missed root canals, file separation, and root canal deviation from the original path, all of which can hinder the clinician's ability to achieve working length [7].

Digital technology, such as cone beam computed tomography and digital impressions, is increasingly used in endodontics to improve treatment planning and simplify procedures. By combining these technologies, dentists can create three-dimensional guides to aid in accessing the tooth during root canal treatment. This can help avoid the complications associated with traditional endodontic surgery [7].

However, traditional guided systems have limitations, including limited visibility, coolant blockage, and challenges in treating posterior teeth [4]. To overcome these challenges, we used the PriciGuide® system, which eliminates the need for a sleeve and improves visibility, coolant access, and maneuverability even in difficult-to-reach areas [5].

Twin Kleen, a hydroxyethylidene diphosphonate-based irrigation solution, was used due to its biocompatibility and decalcifying properties. Hydroxyethylidene diphosphonate can be safely mixed with sodium hypochlorite for brief durations, such as during single-visit endodontics. In contrast to other decalcifying agents, hydroxyethylidene diphosphonate does not aggressively demineralize dentin, helping to maintain its natural integrity [8].

Double antibiotic paste, which contains metronidazole and ciprofloxacin, was utilized as an intracanal medicament because of its antibacterial properties. It was formulated by eliminating minocycline from the triple antibiotic paste to avoid discoloration. Additionally, double antibiotic paste and triple antibiotic paste show comparable antibacterial effects [9].

Internal bleaching was initially considered due to its conservative approach to managing tooth discoloration. However, as satisfactory results were not achieved, direct composite veneers were then considered. Direct composite veneers were fabricated two weeks after bleaching, as the decrease in shear bond strength values is time-dependent [10].

## Conclusions

The PriciGuide® system's advanced technology enabled precise canal location in a calcified maxillary left central incisor. This significantly streamlined the treatment process, ensuring optimal outcomes with minimal errors and chairside time. The conservative approach proved highly effective in achieving predictable results.
